# Nationwide insights into frailty: Systematic review and meta-analysis of community-based prevalence studies from India

**DOI:** 10.1016/j.tjfa.2025.100032

**Published:** 2025-03-11

**Authors:** Sunanda Gupta, Aninda Debnath, Ankit Yadav, Anubhav Mondal, Shweta Charag, Jugal Kishore

**Affiliations:** Department of Community Medicine, VMMC and Safdarjung Hospital, New Delhi 110029, India

## Abstract

Frailty, a biologic syndrome of decreased reserve and resistance to stressors, affects 5 % to 17 % of older adults and is linked to factors like low BMI, female sex, and low exercise levels. With India's older population expected to double by 2050, frailty presents major public health and economic challenges. This study summarizes the prevalence of frailty among community-dwelling Indians. This systematic review and meta-analysis followed PRISMA guidelines to determine the prevalence of frailty among adults in India. We conducted a comprehensive search across multiple databases, including PubMed, Scopus, EMBASE, and Web of Science, up to January 16, 2024, excluding hospital-based studies and reviews. Data were analyzed using STATA software with a random-effects model, and quality was assessed using the JBI Critical Appraisal Checklist. The meta-analysis revealed a pooled frailty prevalence of 36 % (95 % CI: 29 % to 44 %) among 330,007 community-dwelling adults in India, with significant heterogeneity across studies (I² = 99.95 %). Frailty prevalence varied by assessment method, with 48 % using the frailty index and 31 % using the Fried phenotype. Subgroup analyses indicated significant variability in frailty prevalence by gender, data source, and assessment tool, with no significant publication bias detected. This meta-analysis found a pooled frailty prevalence of 36 % and pre-frailty prevalence of 48 % among adults in India, with higher frailty in women (45 %) than men (35 %) and variation across assessment tools. Future research should focus on longitudinal studies and developing tailored frailty assessment tools.

## 1 Introduction

Frailty is defined as “a biologic syndrome of decreased reserve and resistance to stressors, resulting from cumulative declines across multiple physiologic systems, and causing vulnerability to adverse outcomes” [1]. Indicators of frailty encompass simultaneous presence of multiple components like age-related reduction in lean body mass, strength, endurance, balance, walking performance, and low activity [2,3,4,5,6,7,8]. Frailty is a syndrome predominantly seen in geriatric population, affecting 5 % to 17 % of older adults Frailty has been found to be associated with low BMI, female sex, living alone, low levels of exercise, polypharmacy, education, smoking, drinking, malnutrition, and low vitamin D levels, diabetes, hearing dysfunction, cognitive impairment, poor sleep, a history of falls, pain, and depression [9,10].

According to Census 2011, India has 104 million older people (60 years and above), constituting 8.6 % of total population [11]. Experiencing a decadal growth rate of 41 %, the older population in India is anticipated to witness a substantial increase. Projections indicate that the percentage of older individuals in the country is expected to double, reaching over 20 % of the total population by the year 2050 [12]. Frailty assessment scales include the Fried Frailty Phenotype (physical markers), Frailty Index (health deficits), Clinical Frailty Scale (clinical judgment), Edmonton Frail Scale (multidimensional approach), Tilburg Frailty Indicator (self-reported physical, psychological, and social domains), and others. These tools vary in complexity and scope, catering to diverse clinical and research settings. Definition of pre-frailty has also undergone much analysis. Though no consensus has been reached, it has been described as is a multi-dimensional concept, an early and reversible risk-state, before frailty leading to negative healthcare outcomes [13,14]. Systematic review has revealed the prevalence of frailty to be 21 % (15 %−27 %) for physical frailty and 57 % (56 %−58 %) in older population ≥ 50 years of age [15].

Research findings consistently demonstrate a noticeable trend of escalated healthcare expenditures and utilization linked to frailty [16,17]. Studies also unveiled the detrimental economic repercussions associated with frailty status, the components of the frailty phenotype, and the frailty index concerning healthcare costs in older adults [18]. Additionally, upon comparing the Frailty Index (FI) with composite health index of a state (*P* < 0.001), an inverse correlation is observed. In other words, states exhibiting a superior health index tends to have a lower Frailty Index. The elevated prevalence of frailty in low-performing states may be attributed to various factors, including inadequate allocation and utilization of healthcare resources and suboptimal implementation of health policies for the older people [19]. Frailty is also associated with low quality of life [20]. Frailty has proved to be a rising public health issue in recent years [21]. Frailty is a leading health condition among older adults with deep implications for morbidity, mortality, and healthcare needs. Although individual studies have reported prevalence estimates among community-dwelling Indians, variations in methodologies and findings necessitate systematic synthesis. This review attempts to provide a comprehensive understanding of frailty prevalence so that evidence-based interventions and policy planning can be planned in the Indian context. In this study, we aim to summarize available evidence about prevalence of frailty among community-dwelling Indians.

## 2 Materials and methods

The current systematic review and meta-analysis were performed following the PRISMA guidelines. The protocol for this review was registered in the PROSPERO database under the ID CRD42024517520.

### 2.1 Research question and selection criteria

In this meta-analysis, our primary objective was to determine the prevalence of frailty among adults in India, specifically in community-based settings. We included observational studies conducted in India that focused on community-dwelling adults, defined as individuals living independently or with family, **and** excluding those residing in long-term care facilities or institutional settings. Studies based on population survey data, such as the Longitudinal Ageing Study in India (LASI), were also included, as they represent community-dwelling populations on a broader scale [22]. We excluded studies conducted in hospital or institutional settings, review articles, and studies focused on disease-specific populations (e.g., studies restricted to individuals with chronic diseases like diabetes or cancer). Furthermore, studies using convenience samples or those not representative of the general population were excluded to ensure that our findings reflected the broader community context. Only studies involving adults aged 18 years or older were included. Eligible studies were categorized based on the type of data used:

• **Primary data:** Studies where investigators collected original data directly from participants.
• **Secondary data:** Studies utilizing existing sources of population-based survey data, such as LASI.

We included articles written in or translated into English, covering the period from inception to January 16, 2024. Studies were excluded if they involved the wrong study design (e.g., clinical trials, experimental designs, or case studies) or the wrong study population (e.g., populations younger than 18 years, hospital-based cohorts, or disease-specific groups).

### 2.2 Data sources and searches

We performed an extensive literature search across several databases, including PubMed, Scopus, EMBASE, and Web of Science, covering all records up to January 16, 2024, to identify relevant articles for our study. Specific keywords like "frailty," "frail," and "India," along with Boolean operators "AND" and "OR," were used to systematically filter the literature. To enhance search accuracy, MeSH terms, indicated with an asterisk, were also included, as detailed in Supplementary File 1. Our goal was to maintain a broad search strategy to capture as many relevant studies as possible. Additionally, we reviewed the bibliographies of selected articles and related reviews to find any additional pertinent studies. The gathered data were organized using Rayyan.ai software to facilitate the review process and eliminate duplicate entries. The screening process involved an initial title review followed by an abstract examination by two independent reviewers to determine study eligibility. Relevant articles were then managed using Mendeley Desktop V1.19.5 for citation tracking and further detailed analysis. Only studies that met our predefined inclusion criteria proceeded to the data extraction phase.

### 2.3 Quality assessment

The quality of the eligible studies was assessed using the Joanna Briggs Institute (JBI) Critical Appraisal Checklist for Prevalence Studies [23].

### 2.4 Data analysis

For our analysis, we used STATA software (version 18, STATA Corp.) for statistical computations. We presented the combined prevalence rates of frailty and pre-frailty with 95 % confidence intervals (CIs). To account for potential variability among studies, we applied the random-effects model using the restricted maximum likelihood (REML) method. Heterogeneity was measured using the I² statistic and the Chi-square-based Q test. Outliers were identified by a leave-one-out meta-analysis. Subgroup analyses were carried out to explore sources of heterogeneity, categorizing studies by gender, study tool, and year of study. Publication bias was assessed using a funnel plot visualization, with the presence of small-study effects statistically tested through Egger's regression test, setting the significance threshold at *p* < 0.05.

### 2.5 Ethical consideration

This systematic review and meta-analysis consolidated data from previously published studies. The review protocol was registered in PROSPERO to ensure transparency and adherence to predefined objectives and methodologies. We systematically searched and retrieved relevant literature from four major databases. Since our analysis was limited to using published data, ethical approval was not necessary for this review.

## 3 Results

### 3.1 Search and screening results

**Fig. 1:**
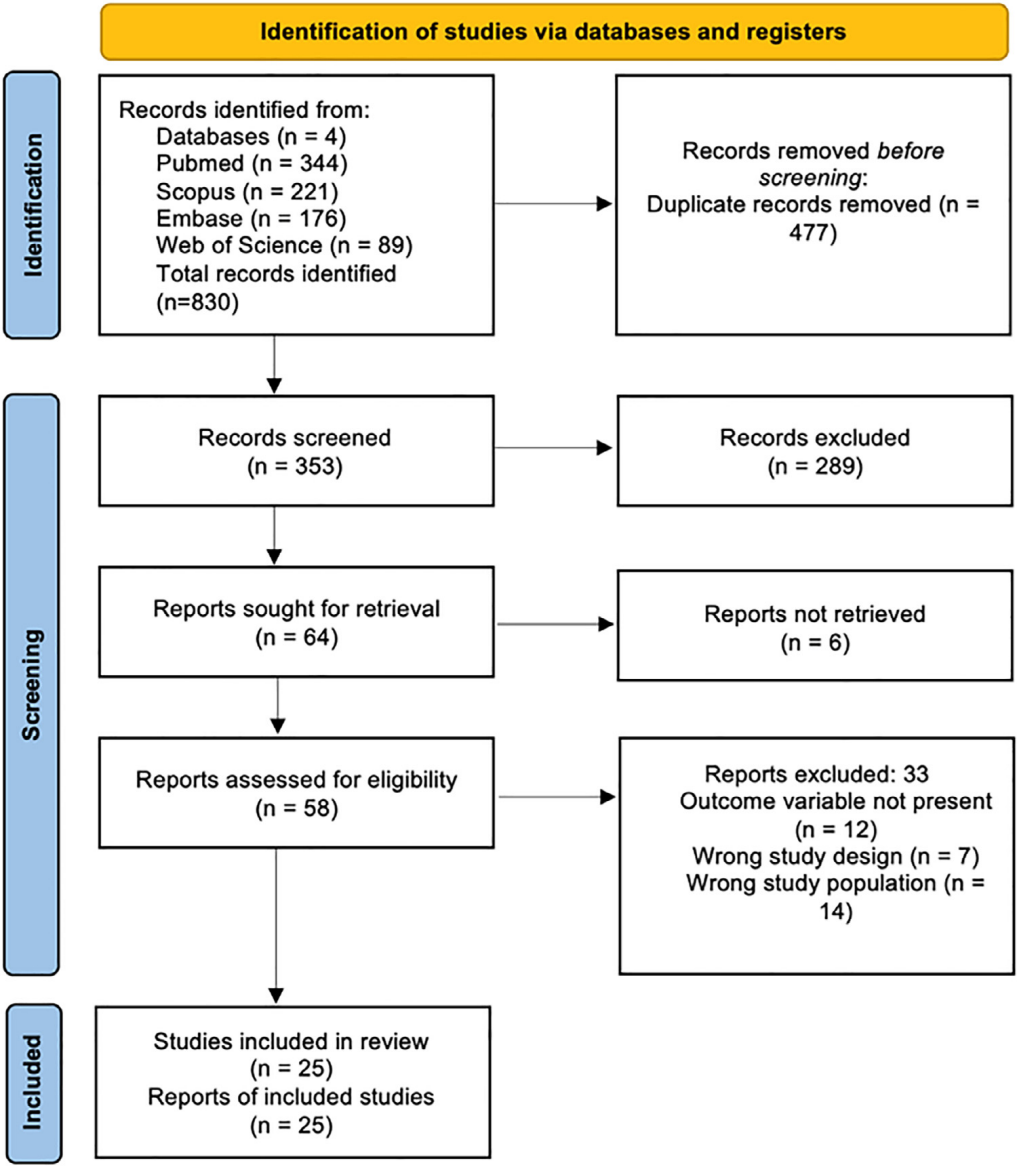
PRISMA flow diagram.

A comprehensive search across four databases yielded a total of 830 articles: 344 from PubMed, 221 from Scopus, 176 from Embase, and 89 from Web of Science. After removing 477 duplicate entries, 353 articles remained. These were initially screened based on title and abstract, resulting in the exclusion of 289 articles. Thus, 64 articles were selected for full-text review. However, full-text versions were unavailable for 6 articles. A thorough eligibility assessment excluded 33 articles that did not meet the predefined inclusion criteria: 12 lacked the outcome variable, 7 had the wrong study design, and 14 focused on the wrong study population. Consequently, 25 articles were included in the final systematic review. The detailed selection process is graphically represented in the PRISMA flow diagram (Fig. 1).

We included 25 studies in our current meta-analysis. Among these, 12 studies used data from nationwide surveys. Specifically, two surveys were used: the Longitudinal Aging Study in India (LASI) and the World Health Organization's Study on Global Ageing and Adult Health (WHO-SAGE). Among the other studies, two were nationwide primary studies, and the remaining studies were from different regions: five from South India, four from East India, and two from North India. Sample sizes varied significantly, ranging from 104 to 57,649 participants [24,25]. The highest prevalence of frailty was noted in the study conducted by Sharma et al., whereas the lowest prevalence was shown in the study conducted by Hoogendijk et al. [26,27], Various tools were used to assess frailty. The most common tool used was the Modified Fried Phenotype, employed in 10 studies. Four studies used the Fried Phenotype, three studies used the Deficit Accumulation Approach and the Edmonton Frailty Scale, and the Tilburg Frailty Indicator was used in two studies each (Table 1). Among the involved studies, 21 (84 %) were of good quality and the remaining 4 (16 %) were of fair quality. No study was found to be of poor quality according to the JBI critical appraisal tool. (Table 2)

**Table 1:** Baseline characteristics of studies included in the meta-analysis.

	Studies	Study tool	Study site	Total Sample Size	Number of participants having frailty n(%)	Number of participants having pre-frailty n(%)	Total female participants n(%)
1	Ghosh et al. [24]	Deficit accumulation approach	India*	57,649	17,006 (29)	–	30,874 (53)
2	Pai et al. [28]	Fried Phenotype	India*	30,390	9090 (30)	19,418 (63)	15,831 (52)
3	Das et al. [29]	Modified Fried	India*	30,978	9023 (29)	–	16,093 (51)
4	Singhal et al. [30]	LASI Frailty index	India*	3953	1769 (45)	–	2130 (53)
5	Seligman et al. [31]	Deficit accumulation approach	India*	52,377	25,806 (49)	20,482 (39)	–
6	Singh et al. [32]	CES-D	India*	31,902	6795 (21)	–	2130 (6)
7	Shilpa et al. [33]	Fried Phenotype	Karnataka	502	124 (25)	315 (62)	228 (45)
8	Kulkarni et al. [25]	Tilburg Frailty Indicator	Karnataka	104	56 (54)	48 (46)	75 (72)
9	Das et al. [34]	Modified Fried	Bengal	510	111 (22)	249 (48)	–
10	Srivastava et al. [35]	Modified Fried	India*	30,551	9800 (32)	–	15,899 (52)
11	Muhammad et al. [36]	Modified Fried	India*	31,464	9644 (31)	–	–
12	Thakkar et al. [37]	Modified Fried	India*	28,285	7782 (28)	–	14,449 (51)
13	Das et al. [38]	Modified Fried	Bengal	510	132 (26)	–	280 (54)
14	Panda et al. [39]	Edmonton Frailty Scale	Delhi	200	76 (38)	51 (25)	104 (52)
15	Rath et al. [40]	Edmonton Frailty Scale	Haryana	834	395 (47)	–	476 (57)
16	Kshatrti et al. [41]	Frailty index for elderly (FIFE) tool	Odisha	725	425 (59)	269 (37)	–
17	Sharma et al. [26]	Linda Fried's Frailty Criteria	Telangana	200	166 (83)	–	124 (62)
18	Shalini et al. [42]	Modified Fried	Telangana	163	33 (20)	–	74 (45)
19	Dasgupta et al. [43]	Tilburg Frailty Indicator	Bengal	165	64 (39)	–	112 (67)
20	Chaudhary et al. [44]	Modified Fried	WHO-SAGE	6560	1277 (19)	3610 (55)	–
21	Kendhapedi et al. [45]	Fried Phenotype	Tamil Nadu	408	104 (25)	201 (49)	232 (56)
22	Chaudhary et al. [46]	Modified Fried	India#	6556	4706 (72)	–	–
23	Hoogendijk et al. [27]	Modified Fried	India#	6372	843 (13)	3070 (48)	–
24	Rodriguez et al. [47]	Fried Phenotype	India	1989	315 (16)	–	1116 (56)
25	Biritwum et al. [48]	Deficit accumulation approach	India	6560	3733 (57)	–	3214 (48)

⁎ = Longitudinal ageing study in India (LASI) wave 1 study.

# = WHO Study on global AGEing and adult health (SAGE).

**Table 2:** Assessment of quality of studies using JBI checklist of critical appraisal for prevalence studies.

	Name of the study	Was the sample frame appropriate to address the target population?	Were study participants sampled in an appropriate way?	Was the sample size adequate?	Were the study subjects and the setting described in detail?	Was the data analysis conducted with sufficient coverage of the identified sample?	Were valid methods used for the identification of the condition?	Was the condition measured in a standard, reliable way for all participants?	Was there appropriate statistical analysis?	Was the response rate adequate, and if not, was the low response rate managed appropriately?
1	Ghosh et al. [24]	Yes	Unclear	Yes	Yes	Yes	Yes	Yes	Yes	Unclear
2	Pai et al. [28]	Yes	Yes	Yes	Yes	Yes	Yes	Yes	Yes	Yes
3	Das et al. [29]	Yes	Yes	Yes	Yes	Yes	Yes	Yes	No	Yes
4	Singhal et al. [30]	Yes	Yes	Yes	Yes	Yes	Yes	Yes	Yes	Yes
5	Seligman et al. [31]	Yes	Yes	Yes	Yes	Yes	Yes	Yes	No	Yes
6	Singh et al. [32]	Yes	Yes	Yes	Yes	Yes	Yes	Yes	No	Yes
7	Shilpa et al. [33]	Yes	Yes	Yes	No	Yes	Yes	Yes	Yes	Yes
8	Kulkarni et al. [25]	Yes	No	No	Yes	Yes	Yes	Yes	Yes	Yes
9	Das et al. [34]	Yes	Yes	Yes	No	No	Yes	No	Yes	Yes
10	Srivastava et al. [35]	Yes	Yes	Yes	Yes	Yes	Yes	Yes	Yes	Yes
11	Muhammad et al. [36]	Yes	Yes	Yes	No	No	Yes	Yes	No	Yes
12	Thakkar et al. [37]	Yes	Yes	Yes	Yes	Yes	Yes	Yes	No	Yes
13	Das et al.[38]	Yes	Yes	Yes	Yes	Yes	Yes	Yes	Yes	Yes
14	Panda et al. [39]	Yes	Yes	Yes	Yes	Yes	Yes	Yes	Yes	Yes
15	Rath et al. [40]	Yes	Yes	Yes	Yes	Yes	Yes	Yes	Yes	Yes
16	Kshatrti et al. [41]	Yes	Yes	Yes	No	Yes	Yes	Yes	Yes	Yes
17	Sharma et al. [26]	Yes	No	Yes	Yes	No	Yes	Yes	Yes	Yes
18	Shalini et al. [42]	Yes	Yes	Yes	Yes	Yes	Yes	Yes	No	Yes
19	Dasgupta et al. [43]	Yes	Yes	Yes	Yes	Yes	Yes	Yes	Yes	Yes
20	Chaudhary et al. [44]	Yes	Yes	Yes	Yes	Yes	Yes	Yes	Yes	Yes
21	Kendhapedi et al. [45]	Yes	Yes	Yes	No	No	Yes	Yes	Yes	No
22	Chaudhary et al. [46]	Yes	Yes	Yes	Yes	Yes	Yes	Yes	Yes	Yes
23	Hoogendijk et al. [27]	Yes	Yes	Yes	Yes	Yes	Yes	Yes	Yes	Yes
24	Rodriguez et al. [47]	Yes	Unclear	Unclear	No	Yes	Yes	Yes	Yes	No
25	Biritwum et al. [48]	Yes	Yes	Yes	Yes	Yes	Yes	Yes	Yes	Yes

#### 3.1.1 Pooled estimate of frailty

**Fig. 2:**
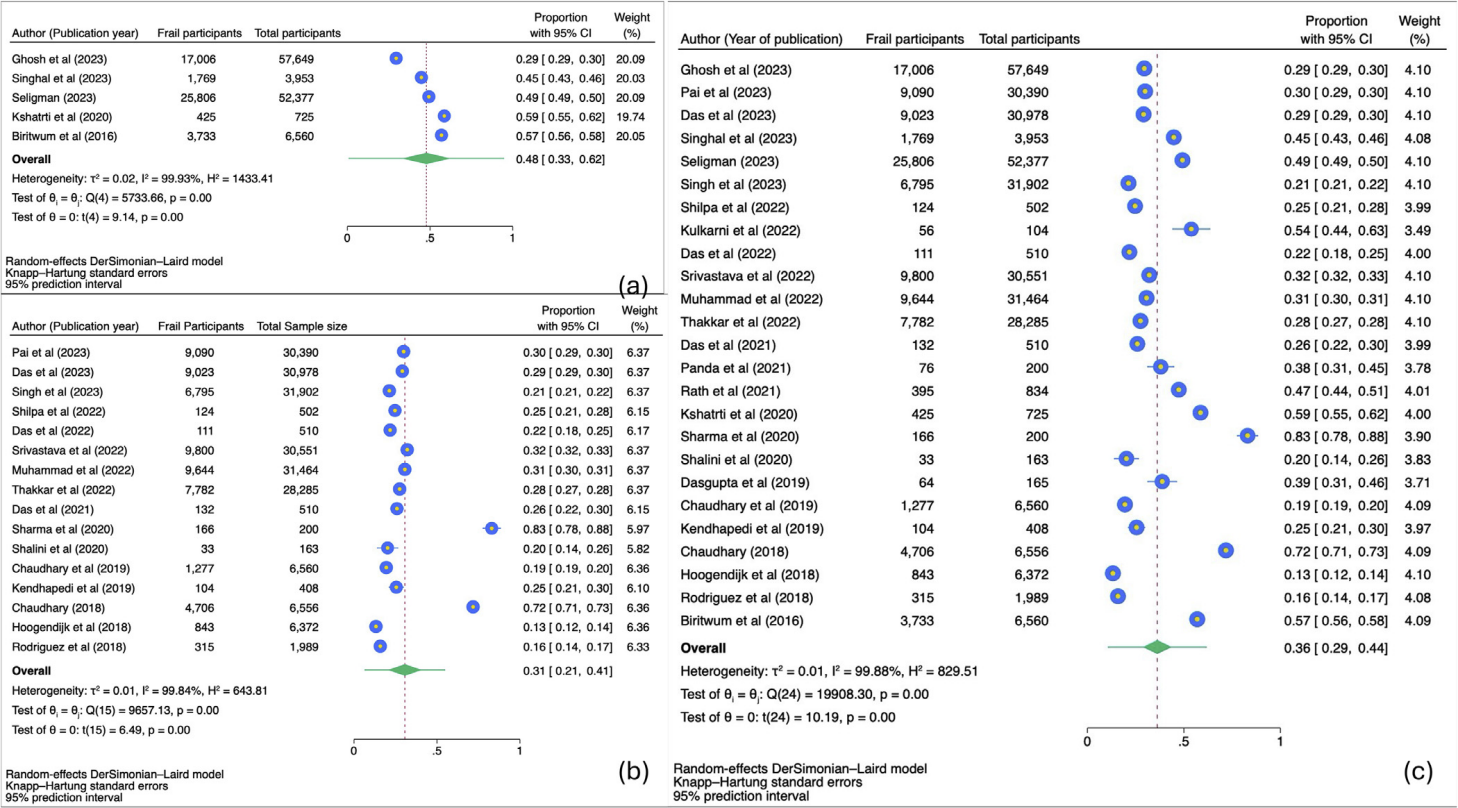
(a) Forest Plot of meta-analysis of prevalence of frailty among community dwelling Indians using frailty index. (b) Forest Plot of meta-analysis of prevalence of frailty among community dwelling Indians using fried phenotype. (c) Forest Plot of meta-analysis of prevalence of frailty among community dwelling Indians irrespective of the tool used to assess frailty.

A comprehensive meta-analysis was conducted to evaluate the prevalence of frailty and prefrailty among community-dwelling adults in India. Frailty was defined using two major methods: the Frailty Index (FI) and Fried Phenotype (FP), and the effect sizes were pooled separately for each method. For studies utilizing the Frailty Index, a total of 121,264 participants were included, among whom 48,739 were identified as frail. The pooled prevalence of frailty in this group was 48 %, with a 95 % confidence interval (CI) ranging from 33 % to 62 % (Fig. 2a). The analysis exhibited substantial heterogeneity (I² = 99.93 %, *p* < 0.01). For studies using Fried's Phenotype, 207,340 participants were analyzed, with 59,945 classified as frail. The pooled prevalence of frailty was 31 %, with a 95 % CI of 21 % to 41 % (Fig. 2b). This analysis also revealed high heterogeneity (I² = 99.84 %, *p* < 0.01). In addition, four studies utilized other methods for defining frailty, and a pooled analysis incorporating all methods was performed. This comprehensive analysis included a total sample size of 329,907 participants, with 109,275 identified as frail. The pooled prevalence of frailty across all studies was estimated at 36 %, with a 95 % CI of 29 % to 43 % (Fig. 2c). Significant heterogeneity was observed in this analysis as well (I² = 99.95 %, *p* < 0.01), highlighting variability in frailty rates across the studies. To account for this variability, a random-effects model was applied in all analyses.

**Fig. 3:**
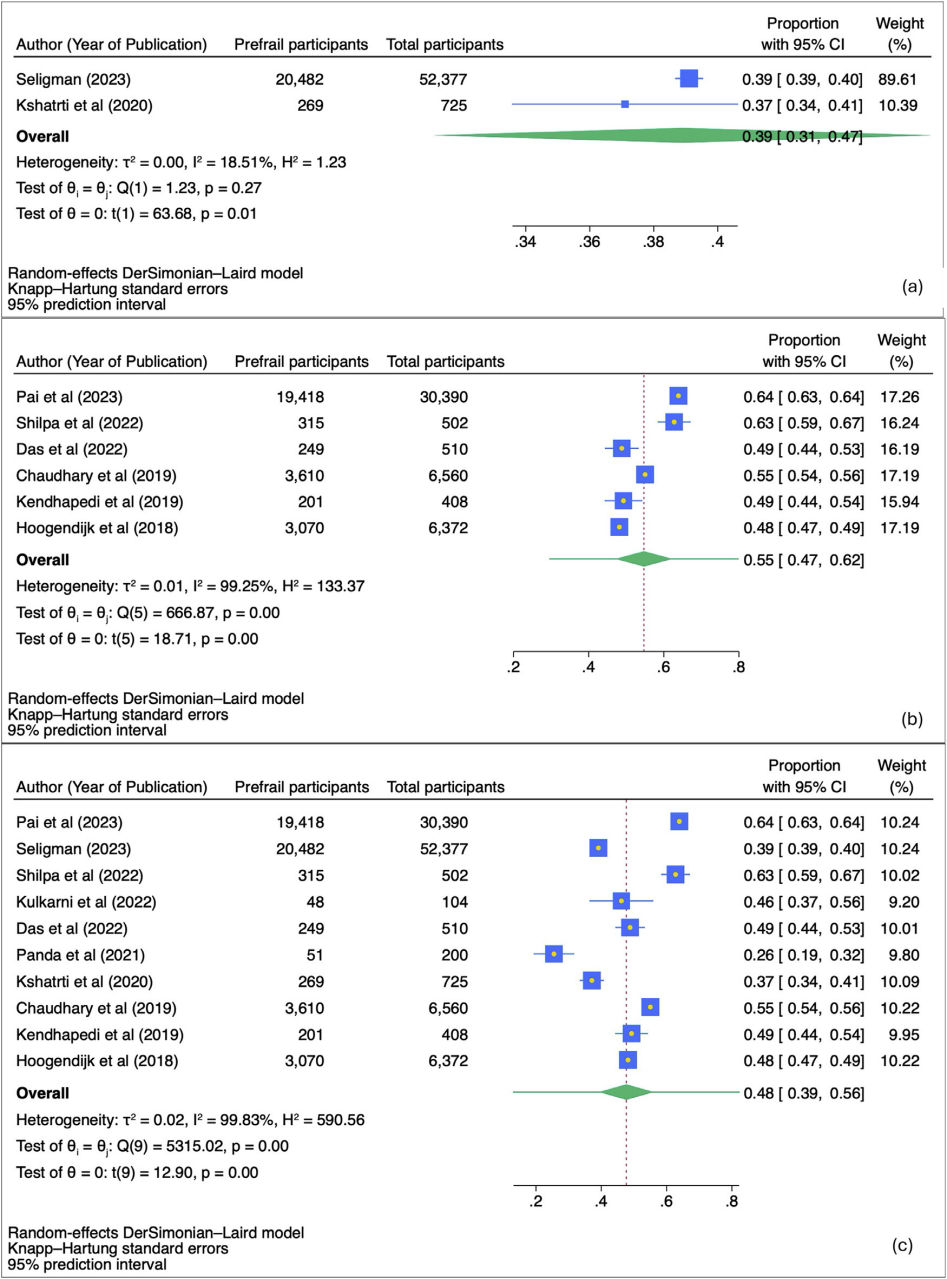
(a) Forest Plot of meta-analysis of prevalence of prefrailty among community dwelling Indians using frailty index. (b) Forest Plot of meta-analysis of prevalence of prefrailty among community dwelling Indians using fried phenotype. (c) Forest Plot of meta-analysis of prevalence of prefrailty among community dwelling Indians irrespective of the tool used to assess frailty.

Fig. 3 presents the meta-analysis of prefrailty prevalence among community-dwelling Indians, using different tools for measurement. Fig. 3a shows that, based on the frailty index, the pooled prevalence of prefrailty is 39 % (95 % CI: 31 % to 47 %) with low heterogeneity (I² = 18.51 %). Fig. 3b reports a higher pooled prevalence of 55 % (95 % CI: 47 % to 62 %) using the Fried phenotype, but with substantial heterogeneity (I² = 99.25 %). Fig. 3c combines data from all studies, regardless of the tool used, showing a pooled prevalence of 48 % (95 % CI: 39 % to 56 %) with significant heterogeneity (I² = 99.83 %). All analyses were conducted using a random-effects model with Knapp–Hartung adjustments.

#### 3.1.2 Publication bias

**Fig. 4:**
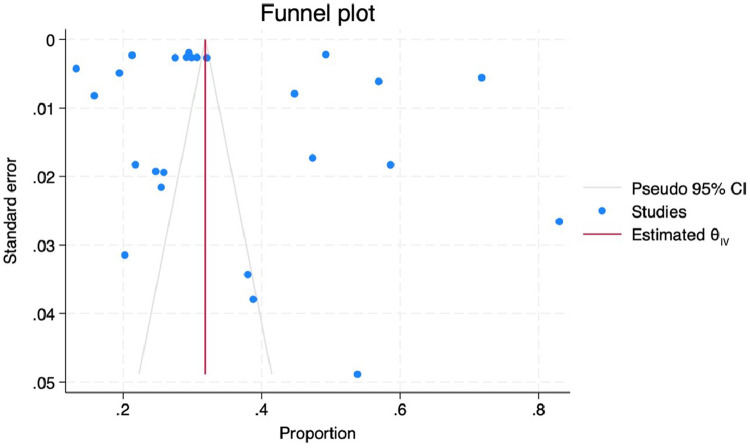
Publication bias.

Although the funnel plot indicated asymmetry, the Egger's test did not provide statistically significant evidence of small-study effects or publication bias (p-value > 0.05). Additionally, the trim and fill method was applied to estimate and adjust for publication bias. This analysis suggested that no additional studies needed to be imputed, indicating that the observed asymmetry might not significantly impact the overall effect size (Fig. 4).

#### 3.1.3 Meta-regression

The meta-regression analyses examined the influence of various covariates, including mean age, sample size, and year of publication. However, none of these covariates demonstrated a significant impact on the effect sizes. [Supplementary file 2–4]

#### 3.1.4 Subgroup analysis

**Fig. 5:**
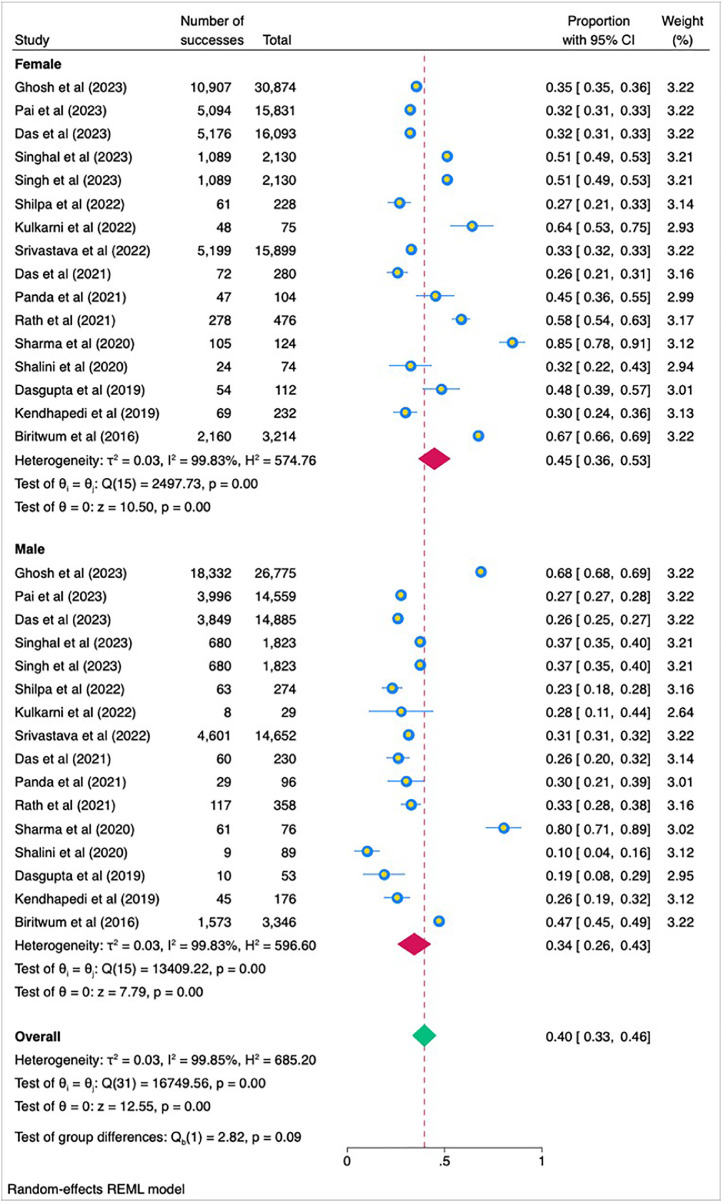
Forest plot showing subgroup analysis according to gender.

To find out the source of heterogeneity we used subgroup analysis based on gender, source of data and study tool. In our meta-analysis, we observed pronounced variability in effect sizes across different subgroups. Gender-based subgroup analysis revealed that for females, the pooled prevalence of frailty was 45 %, with a 95 % confidence interval (CI) ranging from 36 % to 54 %. This subgroup displayed high heterogeneity, with an I² value of 99.85 %, indicating substantial variability across studies. Similarly, for males, the pooled prevalence of frailty was 34 % with a 95 % CI of 26 % to 43 %, and the heterogeneity was also high, with an I² value of 99.85 %. The test of group differences between genders was not significant, suggesting no significant difference in the prevalence of frailty between males and females (Fig. 5).

**Fig. 6:**
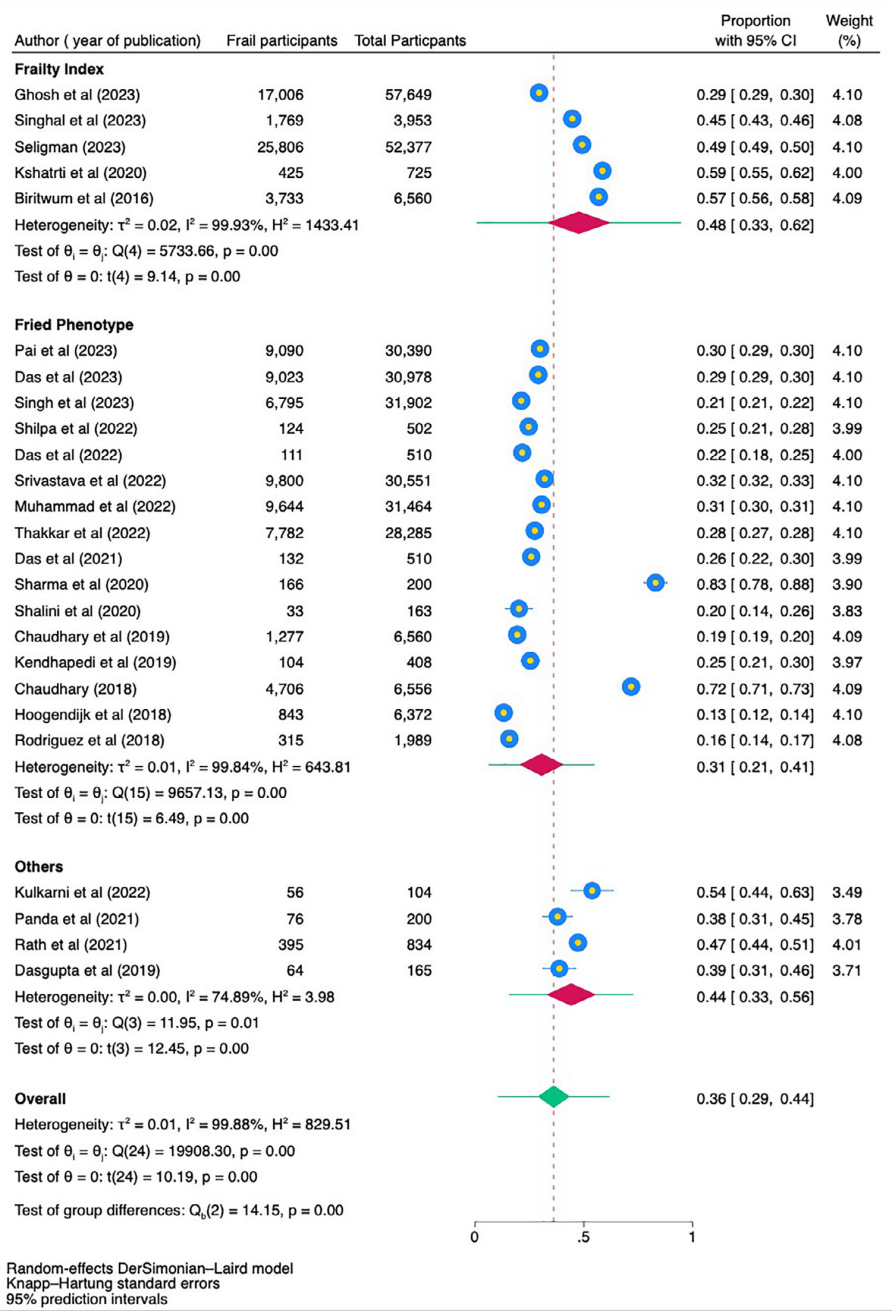
Forest plot showing subgroup analysis based on different tools to assess frailty.

When evaluating frailty measurement tools, the Frailty Index showed a pooled prevalence of 48 % (95 % CI: 33 % to 62 %) with an I² value of 99.93 %, indicating high heterogeneity. The Fried Phenotype reported a prevalence of 31 % (95 % CI: 21 % to 41 %) with an I² of 99.84 %. The subgroup, which includes studies using various other frailty measurement tools, indicated a pooled prevalence of 44 % (95 % CI: 33 % to 56 %) with an I² of 74.89 %, showing moderate heterogeneity. These findings underscore significant variability in frailty prevalence estimates depending on the measurement tool used, and the test of group differences suggests significant variation between subgroups (*p* < 0.01) (Fig. 6).

**Fig. 7:**
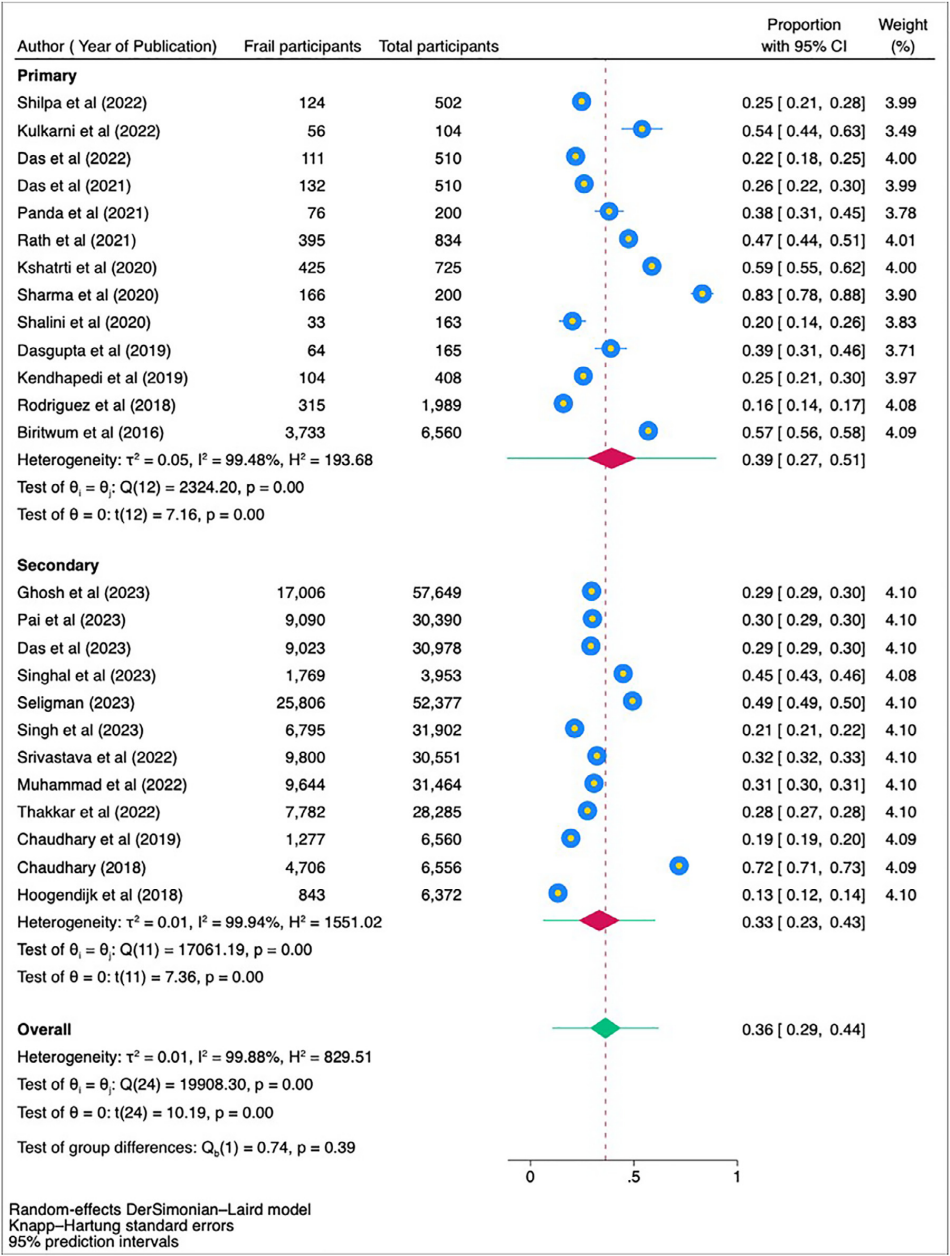
Forest plot showing subgroup analysis based on source of data.

In this subgroup analysis, frailty prevalence was evaluated separately for primary and secondary data sources. The primary data sources showed a pooled prevalence of 39 % (95 % CI: 27 % to 51 %) with an I² value of 99.48 %, indicating substantial heterogeneity. The secondary data sources reported a pooled prevalence of 33 % (95 % CI: 23 % to 43 %) with an I² of 99.94 %, indicating very high heterogeneity. There was no significant difference between the subgroups (*p* = 0.39), suggesting that the source of data (primary vs. secondary) did not significantly impact the pooled prevalence of frailty (Fig. 7).

#### 3.1.5 Sensitivity analysis

**Fig. 8:**
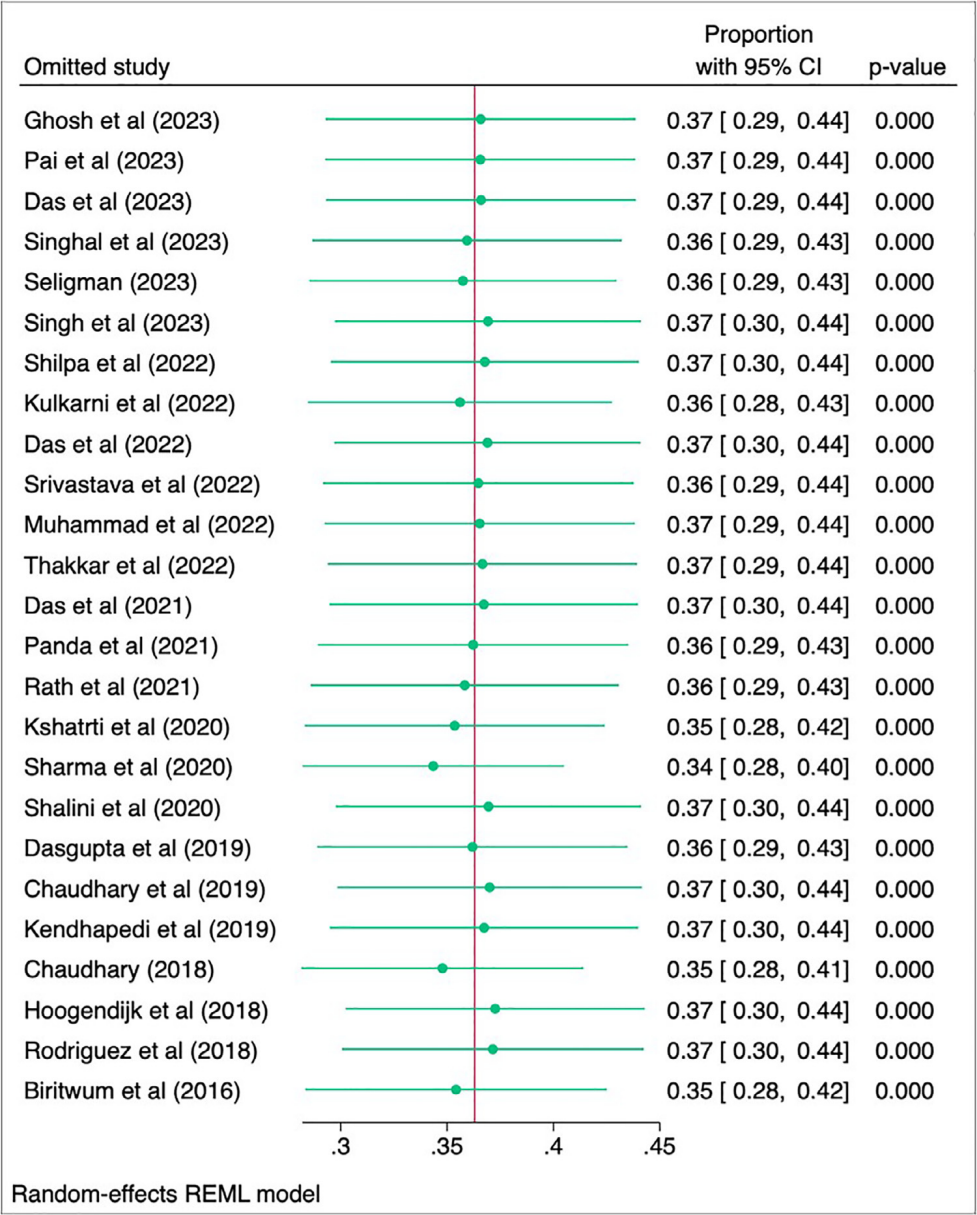
Leave one out analysis.

The leave-one-out analysis and Baujat plot reveal important insights into the robustness and heterogeneity of the meta-analysis results. The leave-one-out analysis shows that the overall effect size, approximately 0.37, remains consistent and statistically significant (*p* = < 0.01) across all iterations, indicating no single study disproportionately influences the overall meta-analytic estimate (Fig. 8). The Baujat plot highlights Sharma et al., Chaudhary et al., and Hoogendijk et al., as studies contributing most to both heterogeneity and the overall result [27,46,26]. When these studies were excluded, the overall proportion slightly adjusted to 34 % (95 % CI: 28 %- 39 %), still statistically significant, and heterogeneity remained high (τ² = 0.02, I² = 99.90 %).(Supplementary 5,6)

## 4 Discussion

A comprehensive meta-analysis was performed to assess the prevalence of frailty and pre-frailty among adults. This study aligns with the rationale by systematically synthesizing diverse evidence to provide a comprehensive understanding of frailty prevalence among community-dwelling Indians, addressing variability in reported estimates. We believe, the findings will assist the development of targeted interventions and health policies to address frailty and promote healthy aging in India's greying population. The pooled prevalence of frailty in this group was 36 %, with a 95 % confidence interval (CI) ranging from 29 % to 44 %. The pooled prevalence of prefrailty is 48 % (95 % CI: 39 % to 56 %). This analysis revealed significant heterogeneity (I² = 99.95 %, *p* < 0.01), indicating substantial variability in frailty rates across the included studies. The variability is accounted to clinical diversity, methodological or statistical diversity [[48], [49]]. The substantial variability in prevalence across studies reflects not only the heterogeneity of measurement tools, such as the Frailty Index and Fried Phenotype, but also the multifaceted nature of frailty, which involves physical decline, cognitive impairment, and social vulnerability. The high heterogeneity indicates that contextual factors—such as regional disparities, socioeconomic conditions, healthcare access, and lifestyle differences—play a critical role in shaping frailty outcomes. To address this, we resorted to random-effect model, sensitivity analysis by leave-one-out method and sub-group analysis [50].

The prevalence of frailty was found to be higher in women, 45 % (CI 36–53 %) than among men, 34 % (CI 26–43 %). This is in line with existing literature [51]. Queuing theory has been used to model deficit accumulation by adapting Little's law, where deficits (L) are seen as the product of environmental stress rate (λ) and recovery time (W). This framework suggests women accumulate more deficits due to higher exposure to stress and slower recovery compared to men [52].

Subgroup analysis revealed the studies using Fried Phenotype scale showed a pooled prevalence of 25 % (95 % CI: 18 % to 31 %). This was lower than the pooled prevalence shown by studies utilizing Deficit Accumulation Approach, Edmonton Frailty Scale, Tilburg Frailty Indicator. However, studies using the Modified Fried approach showed a prevalence of 29 % (95 % CI: 19 % to 39 %). The scales have certain similarities and some dissimilarities. In studies comparing various scales, frailty scores rise nonlinearly with age and showed dose-response relationships with 5-year mortality across all scales. Women consistently had higher frailty scores than men of the same age but exhibited better survival rates compared to men with equivalent frailty scores [53]. Various frailty scores have been proposed without consensus on a gold standard, showing substantial variability in agreement across their mean and between individual pairs. Frailty classification demonstrates wide-ranging agreement levels (Cohen's κ = 0.10–0.83), with the highest agreement among "accumulation of deficits" type scores and the greatest accuracy observed with multidimensional scores, highlighting significant heterogeneity in assessing frailty and identifying frail individuals [54]. Frailty instruments are frequently designed and validated primarily as prognostic tools, but their clinometric properties as evaluative outcome measures remain uncertain [55].

A systematic review exploring prevalence of frailty of sixty-two countries reported that the pooled prevalence for studies using physical frailty measures was 12 % (95 % CI = 11–13 %), compared to 24 % (95 % CI = 22–26 %) for the deficit accumulation model (frailty index). Our study however showed pooled prevalence of 48 % (95 % CI = 33–62 %) for studies using frailty index and 31 % (95 % CI = 21–41 %) for studies using fried phenotype. Pre-frailty prevalence was 46 % (95 % CI = 45–48 %) for physical measures and 49 % (95 % CI = 46–52 %) for FI. Our study revealed pooled prevalence of 39 % (95 % CI = 31–47 %) in studies using frailty index and 55 % (95 % CI = 47–62 %) [15]. The meta-analysis contained data from 62 countries, whereas we reported studies from India. Hence, the results are expected to differ. Other Asian countries reported lower pooled prevalence. Sri Lankan studies estimated frailty to be 15 % (13 %−18 %), Iran reported the prevalence to be 28 % (24 %−32 %) [15].

This study's importance lies in its potential to guide policymakers and healthcare providers in developing targeted interventions, especially as India faces a rapid demographic transition. Understanding frailty at a community level is essential for early identification and prevention strategies, which could delay disability and reduce healthcare costs. Given India's rapid demographic transition, these findings highlight the need for standardized tools to enhance consistency in future research and facilitate more effective resource allocation. Addressing frailty proactively can reduce healthcare costs and improve quality of life, making it a crucial priority in India's evolving healthcare landscape.

### 4.1 Strength

A strength of our study is the robust analysis we conducted. Additionally, we encountered significant heterogeneity during our analysis. To address this, we conducted subgroup analyses, leave-one-out analyses, and used a random-effects model. However, the lack of individual-level data prevented a deeper examination of differences among sample populations.

### 4.2 Limitations

Our investigation recognizes certain limitations. The study did not include grey literature. Furthermore, our abstract screening process may have inadvertently excluded relevant studies where frailty was quantified but not the primary focus, such as those considering it a covariate.

### 4.3 Conclusion

In conclusion, this systematic review and meta-analysis aim to provide a comprehensive overview of frailty prevalence among India's community-dwelling population. By rigorously selecting studies, assessing quality, and synthesizing data using robust statistical methods, we aim to offer a clearer understanding of frailty's epidemiology across diverse regions and populations within India. The findings will inform public health strategies and interventions aimed at reducing frailty burden.

As we conclude this systematic review and meta-analysis on frailty prevalence in India's community settings, several avenues for future research emerge. Firstly, longitudinal studies are warranted to track the trajectory of frailty among different age groups and regions over time. Additionally, investigating the impact of socio-economic factors, lifestyle interventions, and healthcare access on frailty development could provide insights into preventive strategies. Furthermore, there is a need for standardized frailty assessment tools tailored to the Indian population to ensure accurate and comparable prevalence estimates. Lastly, exploring the association between frailty and specific health outcomes, such as disability and mortality, would deepen our understanding of the clinical implications. Addressing these gaps will not only enhance our knowledge base but also support targeted interventions to improve the health outcomes of India's aging population.

## Declaration of competing interest

On behalf of all authors, the corresponding author states that there is no conflict of interest.

## Appendix Supplementary materials

Image, application 1
