# Supplementary material for: Nationwide insights into frailty: Systematic review and meta-analysis of community-based prevalence studies from India
Source: J Frailty Aging. 2025 Mar 11;14(2):100032. doi: 10.1016/j.tjfa.2025.100032 (PMC12183940; doi:10.1016/j.tjfa.2025.100032)

Supplementary file 1:The adjusted search terms as per searched electronic databases [as of 16.01.2024]

| Database | No | Search Query | Results |
| --- | --- | --- | --- |
| EMBASE | | | |
|  | #1 | 'frail elderly'/exp OR 'frail elderly' OR frai*:ab,ti OR frail:ab,ti OR frailty:ab,ti | 60,692 |
|  | #2 | 'india'/exp OR india:ab,ti OR indias:ab,ti | 258,325 |
|  | #3 | #1 AND #2 | 176 |
| PubMed | | | |
|  | #1 | "frail elderly"[MeSH Terms] OR "frail elderly"[MeSH Terms] OR "frail elderly"[MeSH Terms] OR "frail elderly"[MeSH Terms] OR "frail elderly"[MeSH Terms] OR "frai*"[Title/Abstract] OR "frail"[Title/Abstract] OR "frailty"[Title/Abstract] | 42,592 |
|  | #2 | "india"[MeSH Terms] OR "india"[All Fields] OR "india's"[All Fields] OR "indias"[All Fields] | [806,679](https://pubmed.ncbi.nlm.nih.gov/?term=%28India%5BTitle%2FAbstract%5D%29+OR+%28India%29&sort=date&ac=no) |
|  | #3 | #1 AND #2 | 344 |
| Scopus | | | |
|  | #1 | (TITLE-ABS-KEY ( "frail elderly" ) OR TITLE-ABS-KEY ( frai* ) OR TITLE-ABS-KEY ( frail ) OR TITLE-ABS-KEY ( frailty ) ) | [59,568](https://www-scopus-com-aiims.knimbus.com/search/history/results.uri?origin=searchhistory&shid=1) |
|  | #2 | (TITLE-ABS-KEY ( india ) OR TITLE-ABS-KEY ( india's ) OR TITLE-ABS-KEY ( indias ) ) | [601,854](https://www-scopus-com-aiims.knimbus.com/search/history/results.uri?origin=searchhistory&shid=2) |
|  | #3 | #1 AND #2 | 211 |
| Web of Science | | | |
|  | #1 | (((TS=(frail elderly)) OR TS=(frai*)) OR TS=(frail)) OR TS=(frailty) | 43,923 |
|  | #2 | **((TS=(india)) OR TS=(india's)) OR TS=(indias)** | [219,645](https://www.webofscience.com/wos/woscc/summary/dd31a52e-dc4a-4fcd-ba7a-9945f5a34c39-c5bff7d6/relevance/1) |
|  | #3 | #1 AND #2 | 89 |

Supplementary 2: Bubble Plot of Proportion by Mean Age with Inverse-Variance Weights and 95% Confidence Interval


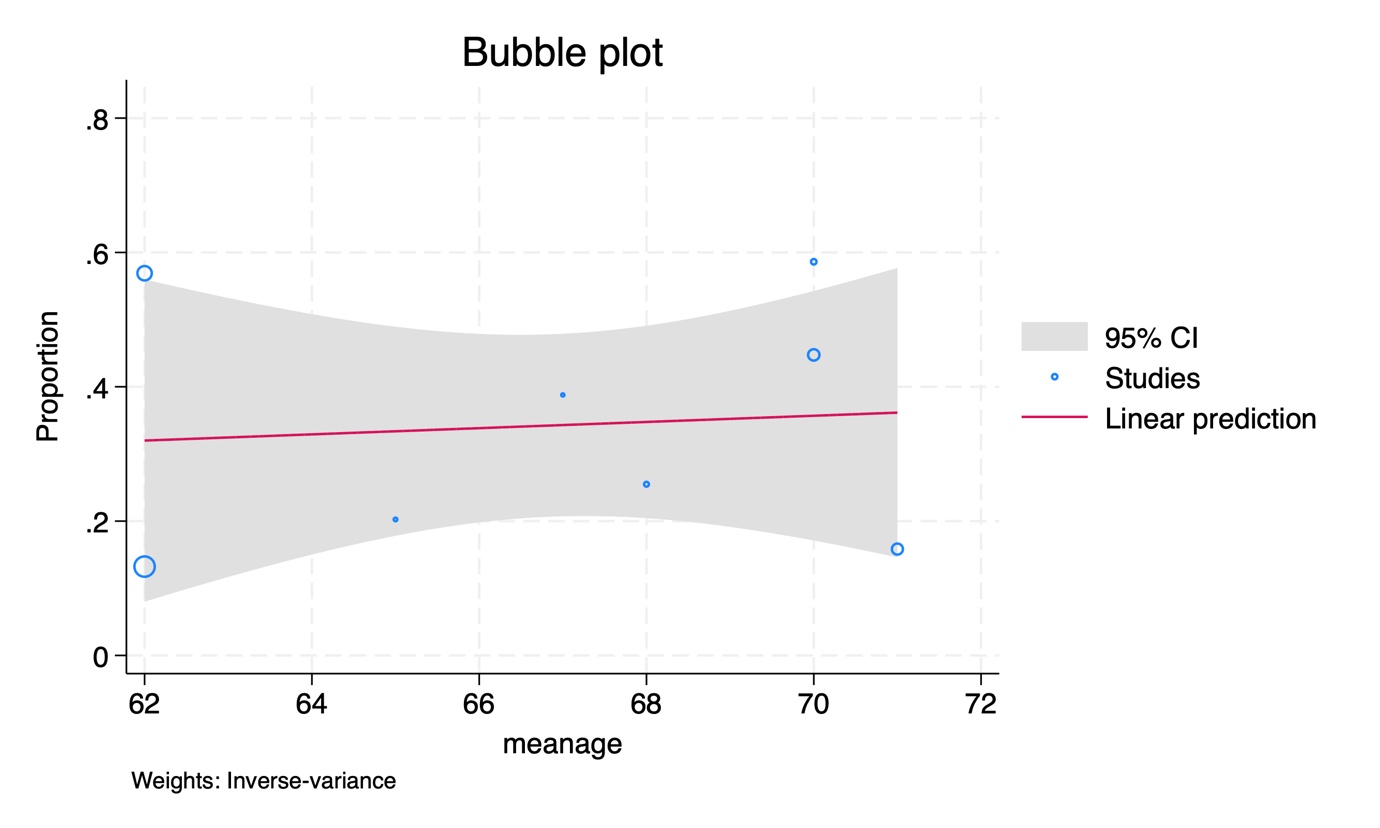


Supplementary 3: Bubble Plot of Proportion by Year of Publication with Inverse-Variance Weights and 95% Confidence Interval


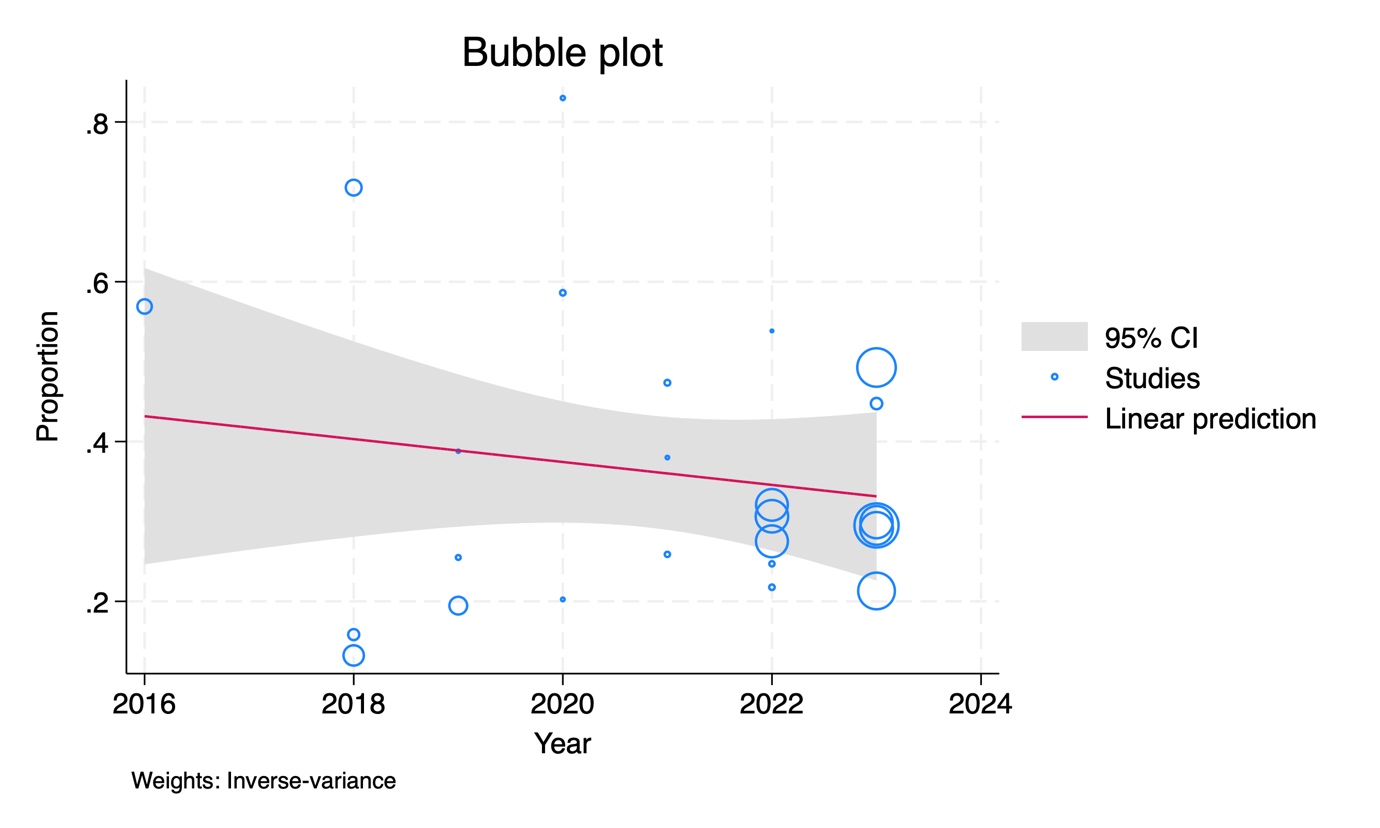


Supplementary 4: Bubble Plot of Proportion by Sample Size with Inverse-Variance Weights and 95% Confidence Interval


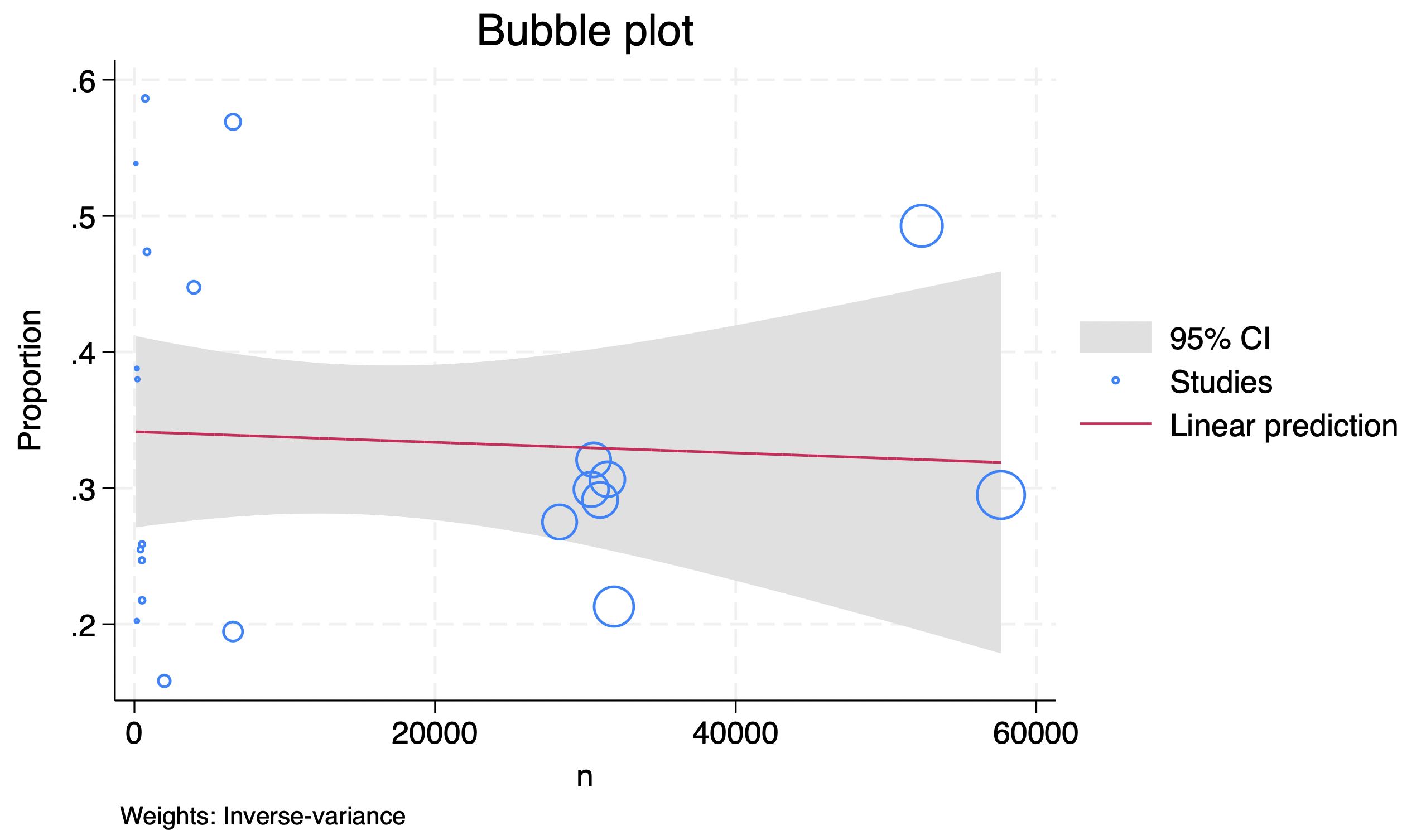


Supplementary 5: Baujat Plot Showing Influence of Each Study on Overall Result and Contribution to Heterogeneity
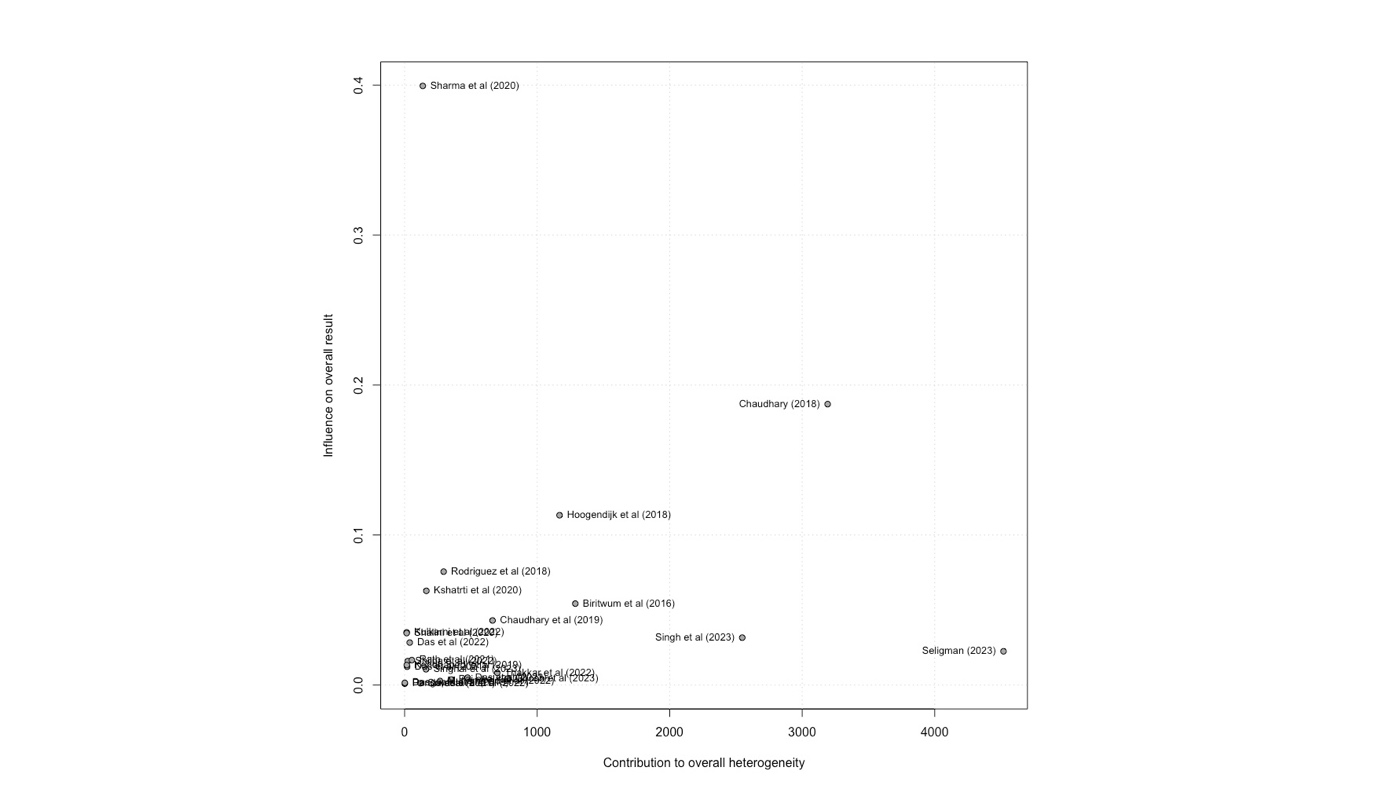


Supplementary 6: Forest Plot of Meta-Analysis Results Excluding Sharma et al. (2020), Chaudhary (2018), and Hoogendijk et al. (2018)


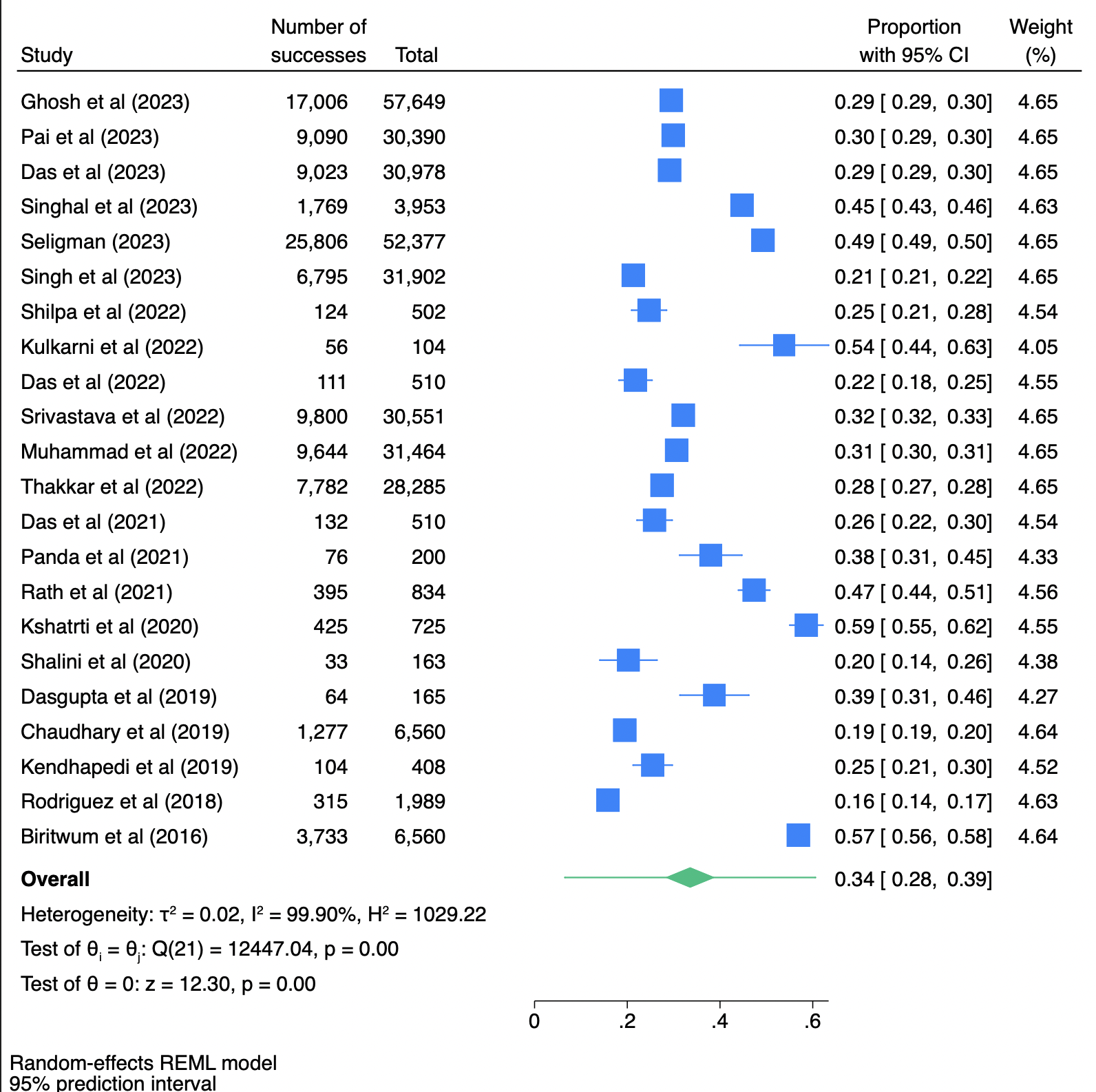

Supplement: Supplementary file 1 [file mmc1.docx]
